# Effect of Filtrated Osmotic Solution Based on Concentrated Chokeberry Juice and Mint Extract on the Drying Kinetics, Energy Consumption and Physicochemical Properties of Dried Apples

**DOI:** 10.3390/molecules26113274

**Published:** 2021-05-28

**Authors:** Klaudia Masztalerz, Jacek Łyczko, Krzysztof Lech

**Affiliations:** 1 Institute of Agricultural Engineering, Wrocław University of Environmental and Life Sciences, Chełmońskiego 37-41, 51-630 Wrocław, Poland; klaudia.masztalerz@upwr.edu.pl; 2 Faculty of Biotechnology and Food Science, Wrocław University of Environmental and Life Sciences, 50-375 Wrocław, Poland; jacek.lyczko@upwr.edu.pl

**Keywords:** osmotic dehydration, filtration, LC-MS/MS, drying kinetics, color, energy consumption, phenolic compounds, chokeberry juice

## Abstract

Background: Filtration of osmotic solution affects selective penetration during osmotic dehydration (OD), and after drying is finished, this can influence the chemical composition of the material, which is also modified by OD. Methods: Osmotic dehydration was carried out in filtrated and non-filtrated concentrated chokeberry juice with the addition of mint infusion. Then, this underwent convective drying, vacuum-microwave drying and combined convective pre-drying, followed by vacuum-microwave finishing drying. Drying kinetics were presented and mathematical models were selected. The specific energy consumption for each drying method was calculated and the energy efficiency was determined. Results and Discussion: The study revealed that filtration of osmotic solution did not have significant effect on drying kinetics; however, it affected selective penetration during OD. The highest specific energy consumption was obtained for the samples treated by convective drying (CD) (around 170 kJ·g^−1^ fresh weight (fw)) and the lowest for the samples treated by vacuum-microwave drying (VMD) (around 30 kJ·g^−1^ fw), which is due to the differences in the time of drying and when these methods are applied. Conclusions: Filtration of the osmotic solution can be used to obtain the desired material after drying and the VMD method is the most appropriate considering both phenolic acid content and the energy aspect of drying.

## 1. Introduction

Osmotic dehydration (OD) is a process that occurs when a material is immersed in hypertonic solution and as a result, due to the osmotic pressure between the solution and the material, mass transfer occurs. Osmotic dehydration used to be carried out in salt or sugar solutions [1,2], but nowadays, the use of osmotic solutions based on concentrated fruit or vegetable juices is gaining recognition [3,4,5,6]. The application of concentrated juices results in better characteristics of the dehydrated material as the substances from the juice flow into the material, improving its properties [7,8,9]. Yet, the use of this type of osmotic solution comes with some limitations and might disrupt the process due to the heterogenous composition of the juice, which contains a broad spectrum of different molecular weight substances [3].

The size of the particles determines the intensity of the process as the smaller molecular weight ones are faster to move and could penetrate deep into the material [10], which is consistent with the Stokes–Einstein Law, which explains the relation between molecular weight and diffusion in liquids and shows that the smaller the molecular weight the faster the diffusion [11]. Conversely, bigger particles tend to be stuck on the surface of the material, hindering mass transfer, which was observed in previous studies by Goula et al. [12], who performed osmotic dehydration of potatoes in sodium chloride and maltodextrin solutions, and Antonyuk et al. [13], who studied the issues concerning agglomeration of particles of different molecular weight. A high variety of different size particles in concentrated juice is strictly associated with the type of phenolic compounds present in the juice and its antioxidant activity, as reported by Lech at al. [3]. Another important factor is size of the openings in the material, which can also significantly affect the osmotic dehydration [14]. Therefore, filtration of concentrated juice can be successfully applied to remove bigger particles that can hinder the process, and consequently, facilitate the OD, which was previously described by Masztalerz et al. [15], where filtrated chokeberry juice was used as osmotic solution during osmotic dehydration of pumpkin. That study revealed that application of filtrated solutions resulted in an even distribution of solution particles in the material as well as leading to higher antioxidant capacity when filtrated OS was used.

Filtration can be also used to recognize the selective penetration phenomena occurring inside the material when concentrated fruit juice is applied as an osmotic solution.

Osmotic dehydration is one of the drying methods that is usually applied as a pretreatment due to the relatively high moisture content after the process. Therefore, osmotic dehydration is frequently followed by other drying methods, such as convective drying, vacuum-microwave drying and others [16,17,18]. However, drying can have a negative effect on the volatile composition of dried biological material, as reported in the studies on Thai basil that was dried using convective (CD), vacuum-microwave (VMD) and combined drying (CD-VMD) and showed that drying resulted in a significant reduction of the number of total essential oils in comparison to the fresh material [19]. Additionally, drying often results in lower antioxidant activity and polyphenolic content, which was presented in a study on Pink Rock Rose [20], which reported a decrease in the bioactive potential as a result of long oxidation and elevated temperatures. Yet, selection of appropriate drying methods and parameters can reduce the negative effects of drying [21,22].

Energy efficiency and energy consumption can also be an important factor influencing the choice of drying method. Although the process on a laboratory scale is much less effective than when conducted on commonly used industrial equipment, the tendencies and characteristics of each method are similar in both cases. Several factors affect the energy consumption during drying, e.g., the type of surface of the material [23], temperature of drying [24] and others. As previously described, the applied drying method also significantly affects the energy consumption during the process, with lower energy consumption during vacuum-microwave drying (when dried at 480 W: 78.2 kJ·g^−1^ fw and 48.9 kJ·g ^−1^ fw for arils and rind, respectively) compared to convective drying (228 kJ·g^−1^ fw and 229 kJ·g^−1^ fw for arils and rind, respectively) in the case of pomegranate arils and rind [25], which was strictly connected with duration of the process. Combined methods, consisting of convective pre-drying and vacuum-microwave finishing drying, were also previously considered [26] and this combination of different drying methods significantly reduced the time of drying, and therefore, the specific energy consumption (energy savings in the range from 54.42% to 86.25% in respect to convective drying) and led to high-quality dried materials [27]. However, to our knowledge, there are no studies on energy consumption during osmotic dehydration followed by different drying methods when processing of apple cylinders was considered.

Considering mentioned issues, osmotic dehydration in filtrated and non-filtrated osmotic solutions based on concentrated chokeberry juice with the addition of mint infusion was conducted in the study. The process was followed by convective drying, vacuum-microwave drying and a combined method consisting of convective pre-drying and vacuum-microwave finishing drying. The main aim of the study was to assess the effect of the filtration of osmotic solutions on finishing drying and on phenolic acids, originating from mint infusion and concentrated chokeberry juice, both in the material and osmotic solution. Additionally, the specific energy consumption as well as efficiency of water evaporation of each drying method were considered and presented.

## 2. Results and Discussion

### 2.1. Osmotic Dehydration

Solid gain (SG), water loss (WL) as well as the water loss to solid gain ratio (WL/SG) and specific energy consumption during drying (*E_ODw_* and *E_ODm_*) are presented in Table 1. Preliminary studies and a literature review revealed that the most intense mass exchange occurs at the beginning of the process, and therefore, osmotic dehydration was carried out for 30 min [15]. Due to the application of heterogenic concentrated chokeberry juice with the addition of mint infusion, osmotic solutions were filtrated to remove high molecular weight compounds that could disrupt the mass transfer. As a result, OD in filtrated osmotic solution (0.2 OS; filtrated using filter with pore size 0.2 µm) resulted in only slightly higher WL than in the case of non-filtrated osmotic solution (NF OS). As for SG, there were no significant differences between the variants.

Energy consumption during OD was equal to 126 kJ for both variants; however, specific energy consumption expressed as kJ·g^−1^ water was higher for NF OS, which is due to the lower WL when this variant was applied. Nonetheless, osmotic dehydration is a complex process. The aim of OD carried out in concentrated chokeberry juice with the addition of mint extract was to obtain the highest value of solid gain. Therefore, unit consumption of energy needed for evaporation of 1 g of water was not crucial in the case of osmotic dehydration.

### 2.2. Drying Kinetics

**Figure 1:**
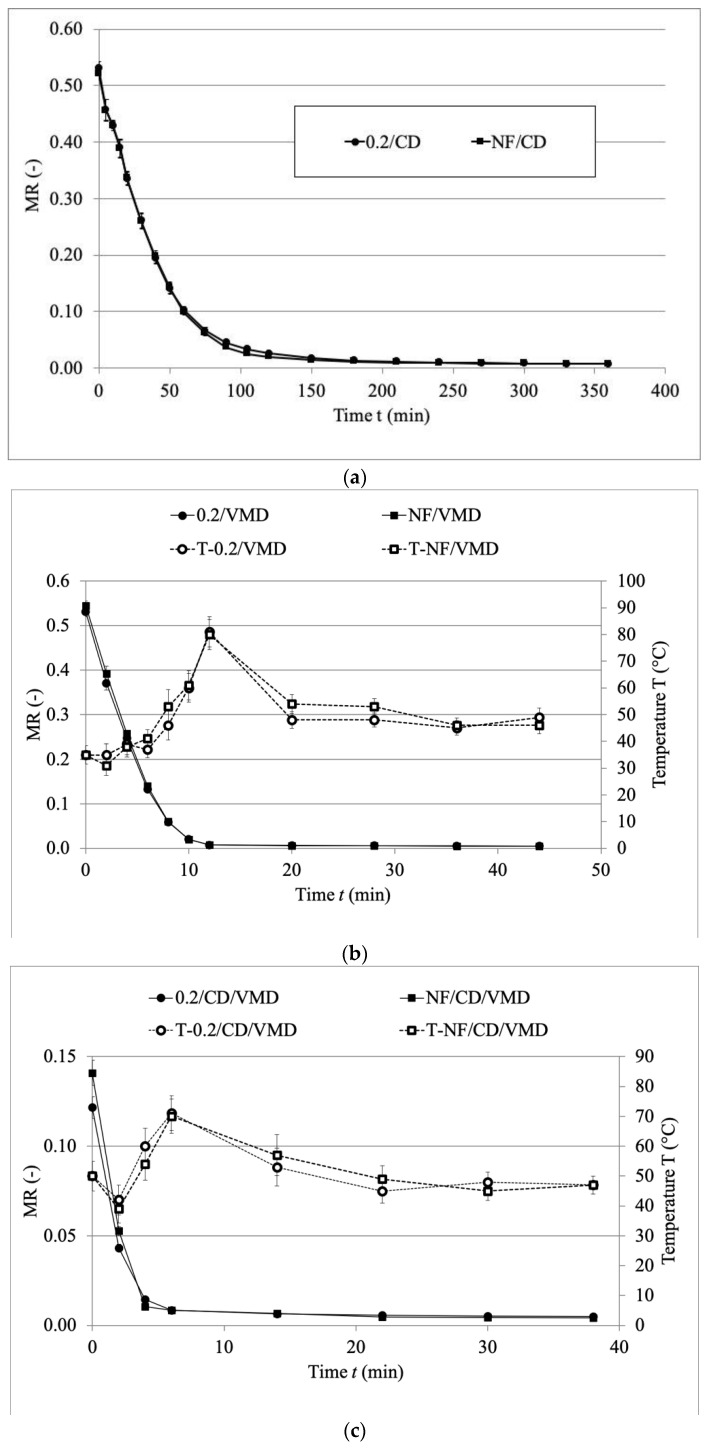
Drying kinetics of the samples dehydrated in filtrated and non-filtrated solutions and drying using (**a**) convective drying, (**b**) vacuum-microwave drying, (**c**) combined drying: convective pre-drying and vacuum-microwave finishing drying. T—series starting with this letter refer to temperature profile of the material during drying.

The drying kinetics of the samples treated by different drying methods are presented in Figure 1. Filtration of the osmotic solution had no effect on the drying kinetics regardless of the applied drying method. However, the drying kinetics varied between the methods. In the case of VMD, the process was much more intensive compared to CD, which is shown by higher values of drying constant *k* (Table 2). When VMD was applied, transformation of microwave energy into heat occurred, leading to volumetric heating. As a result, high vapor pressure built up inside the dried material due to the pressure diffusion mechanism of the Darcy type, which is more intensive than conventional solute diffusion, as stated in Fick’s Law [28]. Therefore, the time of drying of the samples treated by VMD was significantly shorter than in the case of CD—over 280 min in the case of CD and 12 and 13 min in the case of 0.2/VMD and NF VMD, respectively (Table 3).

When combined drying was applied, unbound water was removed in the convective pre-drying part and following vacuum-microwave finishing drying resulted in the removal of residual moisture that was bound to the plant tissue [29]. Consequently, the time of the process was reduced from over 280 min for CD and over 12 min for VMD to 60 min of CD and as little as 5 min of VMD in CD/VMD variants. Similar findings were reported in the studies on True Lavender [30], with significantly lower values obtained when the combined drying method was applied compared to solely using CD. This is due to the different drying mechanisms when these methods are applied, but can also be caused by the molecular distribution of water particles inside the material when CD is applied, which facilitates the heat generation during the second stage of drying when VMD is applied [31].

Temperature is one of the crucial factors affecting the quality of the final product [32]. The temperature of the sample during CD was equal to the temperature of the drying air at the end of the process and amounted to 60 °C. On the other hand, the temperature during VMD (Figure 1b, series T-NF/VMD and T-0.2/VMD) ranged from 35 °C to 80 °C, as the material was subjected to osmotic dehydration prior to drying which resulted in filling up of the pores inside the sample that led to disrupted water removal during VMD, and therefore, heating up of the material [33]. However, it is worth noting that the real temperature inside the sample was much higher than the surface temperature measured by the infrared camera. To avoid overheating, the power of the magnetrons during VMD was reduced from 480 W to 120 W when the temperature rose above 70 °C (Table 3). A similar procedure was used in the case of vacuum-microwave drying of pumpkin [34], where the power of the magnetrons was reduced from 480 W to 120 W when the temperature reached 80 °C.

The time of power reduction (*t_R_*), time of drying (*t*) and time of reaching a moisture content equal to 5% (*t_5%wb_*) is presented in Table 3. As can be seen, the power of the magnetrons was reduced from 480 W to 120 W after the sample reached 5% in most cases (except NF/VMD), and further drying of the material resulted in over six times longer drying in the case of the combined method and over three times longer in the case of VMD.

Three mathematical models were considered in the study (Table 2) and each one of them resulted in a very high fit, expressed as high R^2^ (R^2^ > 0.9777 for each variant), and low values of RMSE (RMSE < 0.0262). The best fit for samples treated solely by OD and CD as well as OD and VMD was obtained for Modified Page’s model, which was previously used to describe the experimental data in the studies on true lavender leaves [30] and peppermint [35]. In the case of combined drying, only the VMD finishing drying was modeled and the best fit was obtained for the logarithmic model, which could be due to the more complex form of this model. The logarithmic model was previously used in studies on sweet potato [36] and mint leaves [37]. All of the applied models included the drying coefficient *A*, which represented the starting point for drying. In this case, the value of the *A* coefficient depended on the moisture content obtained after osmotic dehydration, and in the case of combined drying, showed an MR value after osmotic dehydration and convective pre-drying. A lower value of the *A* coefficient in other drying variants is a result of higher WL during osmotic dehydration. Drying constant *k* corresponded with the time of drying, with a shorter time obtained when *k* was higher, which is consistent with the studies on beetroot slices [33]. In most cases, *k* was lower for the samples treated by the non-filtrated solution. Filtration of osmotic solution facilitated the mass transfer due to the removal of high molecular weight particles that could impregnate the outer layer of the material, leading to lowering of the permeability of the plant tissue, which is consistent with the studies by Lenart and Lewicki [38], therefore resulting in lower *k* for NF variants.

Overall, the modified Page’s model proved to better describe the experimental data obtained in the study.

### 2.3. Color

Color is one of the most important parameters that shape the perception of food by consumers, and as such, needs to be carefully monitored and evaluated. Lightness (L*), redness (a*) and yellowness (b*), along with a total change in color (∆E*), are presented in Table 3. L* values ranged from 21.55 in the case of O.2/VMD to 25.49 in the case of 0.2/CD, while the fresh sample amounted to 71.16. Overall, lighter samples were the ones dried by CD, which is due to the lower temperature of convective drying, whereas during VMD, the sample was heated up to 80 °C. The lowest a* value was obtained for NF/CD samples (8.58), while NF/CD/VMD reached 16.86. Fresh sample showed negative value (−2.46), which means that the fresh material was greener. Drying methods did not affect the b* parameters. Similar values were obtained in the studies by Lech et al. [14], where apples were subjected to OD in concentrated chokeberry juice. ∆E* is the total color difference determined in relation to the fresh material and can be classified as very distinct (∆E* > 3), distinct (∆E* = 3–5) and small difference (∆E* < 1.5) according to Adekunte et al. [39]; however, according to Pérez-Magariño and González-Sanjosé [40], the human eye can only distinguish two samples when *∆E* > 5. Very high values of ∆E* were the result of osmotic dehydration in chokeberry juice, which changed the color of the sample as the red pigment from the juice was absorbed by the apple tissue. OD in concentrated juices was also previously reported to change the color of the dried material to that of a juice in the studies on pumpkin [34], which can be beneficial as this type of osmotic solution can be used to conceal the discoloration occurring in the biological material during drying and can also be used to modify the color of the dehydrated material using natural concentrated juice.

### 2.4. Phenolic Compounds

The phenolic acids found in the dried apples and osmotic solutions are presented in Table 4. When considering the effect of filtration on phenolic acids inside the material, only the content of caffeic acid (CA) showed higher values when filtrated solution (0.2 OS) was applied (Table 4). This effect can be associated with the size of the particles taking part in the process when 0.2 OS was used. Filtration of osmotic solution not only removed high molecular weight particles from the solution, but also affected the selective penetration during the process. Smaller particles were faster to move and could penetrate further into the sample resulting in better retention of bioactive compounds during drying. This hypothesis was supported by the CA, RA and ChA particle size approximation obtained by modeling with graphical tools available in sophisticated software (HyperChem). The approximated particles sizes (including the most optimal particles geometry) for CA, RA and CHa were 0.8 nm, 1.1 nm and 1.3 nm, respectively. This is consistent with the amount of CA in osmotic solutions before and after 30 min of the process. Reduction of CA in the case of NF OS before and after OD was much higher (by 43%) than in the case of 0.2 OS (by 15%). However, samples treated by NF OS exhibited lower values of CA than in the case of 0.2 OS, which can be due to the size of the particles taking part in the process when filtrated solution was applied. This is consistent with the previous studies by Masztalerz et al. [15], who reported significantly higher antioxidant activity of dehydrated apples when filtrated osmotic solution was applied.

The contents of rosmarinic acid (RA) and chlorogenic acid (ChA) in the material were similar for the samples dehydrated in filtrated and non-filtrated solutions, but were significantly (*p* < 0.05) affected by the drying method. The lowest values of RA were obtained in the case of CD, and almost two times higher values were obtained when other methods were applied, which can be due to the oxygen exposure when this method was applied. Mint infusions are known to contain high content of phenolic compounds such as rosmarinic acid [41], while chlorogenic acid can be found in great quantities in concentrated chokeberry juice [3]. The use of CD resulted in significantly higher amounts of ChA, with the highest value obtained in the case of NF/CD. This can be due to filtration that partially removed higher molecular weight compounds and resulted in lower content of ChA in filtrated solution, which is inconsistent with the studies by Lachowicz et al. [42], who reported that filtration of chokeberry juice did not affect the amount of chlorogenic acid. However, in our study, chokeberry juice was diluted to 20 °Brix before filtration and then evaporated to concentrate the juice to 40 °Brix, which could be the reason for the initial lower content of ChA in filtrated OS (0.2 OS), and therefore, lower content of this phenolic acid in the material after drying.

Two times higher initial content of RA was found in NF OS compared to filtrated solution (0.2 OS) before OD. Yet, samples treated by 0.2/VMD and 0.2/CD/VMD contained higher amount of RA after drying than the variants treated by NF solution, which suggest that filtration facilitated the transport of RA into the material. Additionally, samples dried by VMD and CD/VMD did not show any significant differences and contained a higher amount of RA compared to CD variants as well as lower ChA values than CD.

Overall, filtration of osmotic solution influenced the content of phenolic acids both in osmotic solution and material after drying. However, the applied drying method affected the phenolic acids content in the material to a greater extent than filtration of OS.

### 2.5. Energy Consumption during Drying

**Figure 2:**
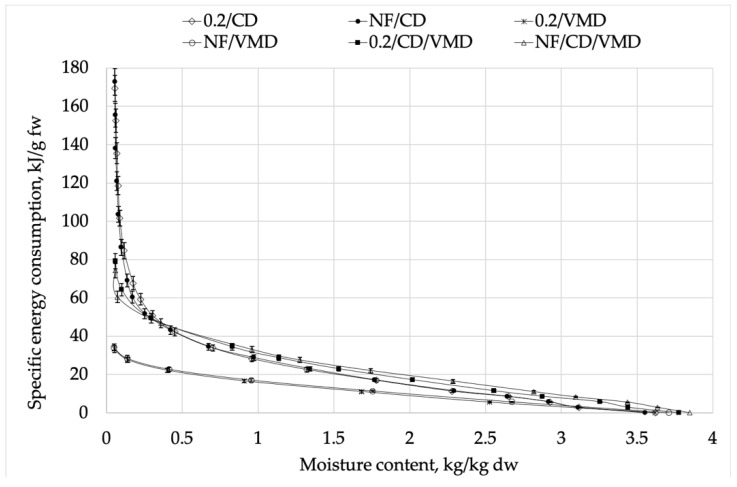
Specific energy consumption during drying using different methods.

Specific energy consumption presented as kJ·g^−1^ fresh weight is shown in Figure 2. The final moisture content varied between the variants; therefore, the drying kinetics were used to determine the time of obtaining 5% moisture content *t_5%eb_*, which is presented in Table 3, and thus, the specific energy consumption was calculated regarding the time of obtaining the same value of *M_C_*.

Specific energy consumption at the beginning of the process (Figure 2) was very low, and subsequently, increased along with the moisture content reduction, with a spike in energy consumption at the last stages of drying, which is typical for the plant materials with osmotic cellular structure [43]. This phenomenon can be associated with the states of water inside the biological material. At the beginning of drying, mostly easily accessible unbound water is removed; therefore, the energy needed for evaporation is relatively low. Then, more energy is needed to remove the bound water from the material, which is supported by previous studies [44].

The highest specific energy consumption was obtained for CD variants and the lowest specific energy consumption was obtained for VMD. Similar results were reported in the studies by Stępień et al. [20]. Application of combined drying resulted in significant reduction (by over 50%) of specific energy consumption compared to the sole use of convective drying, from around 170 kJ·g^−1^ fw for CD to around 70 kJ·g^−1^ fw in the case of combined drying. However, VMD still needed lower energy, which can be associated with the shortest time of drying among all methods. This is consistent with work by Calín-Sánchez et al. [45]. Considering the data shown in Figure 2 and Table 3, it can be concluded that with shorter time required to reduce moisture content to 5%, the energy consumption decreased, which is consistent with previous studies by Zambra et al. [46] and Amado et al. [47].

**Figure 3:**
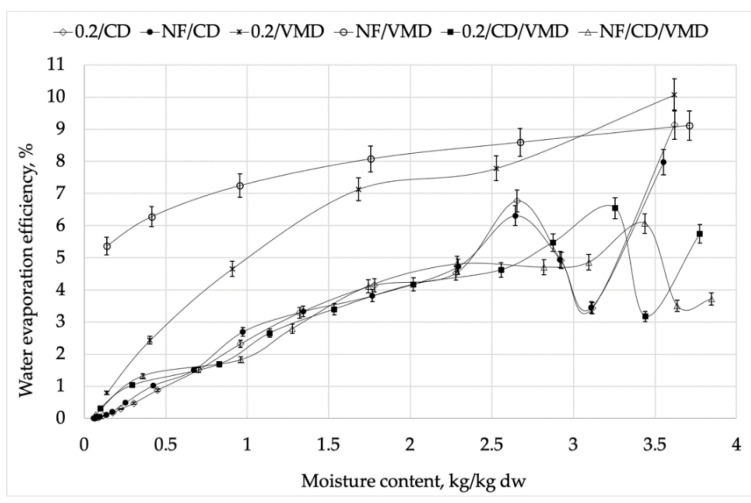
Energy efficiency during drying using different methods.

In the case of CD as well as combined drying (CD/VMD), relatively high initial efficiency of water evaporation (Figure 3) can be seen, which was a result of the drying of external moisture that remained after osmotic dehydration. Even though the external moisture was gently removed by paper towels immediately after OD, some moisture still remained and was easily evaporated at the beginning of convective drying, resulting in relatively high energy efficiency (4–9%). Then, a decline in energy efficiency can be reported, followed by a further increase. This drop can be associated with the heating up of the material; thus, the energy input was mostly utilized to increase the temperature of the material rather than water evaporation. This is natural for convective drying, where hot air flows through the sample and water reduction occurs from the outer layers of the material. A gradual decrease of energy efficiency after the initial period was connected with an increase of specific energy consumption (Figure 2).

Higher water evaporation efficiency (9–10%) when the VMD method was considered came from the drying mechanism typical for this method when the material was heated up in a whole volume, and therefore, higher values of drying constant *k* were reported (Table 2). Additionally, the use of reduced pressure during VMD led to a building up of the pressure gradient, which contributed to accelerated water transport when this method was applied. Time of drying was significantly reduced, and therefore, more water could be evaporated in a shorter time, consequently leading to higher energy efficiency. It is worth noting that the efficiencies obtained in the study (below 10%) were calculated for laboratory scale equipment and drying occurred in single layer; therefore, the presented values were substantially lower compared to processes carried out on a industrial scale. However, the tendencies and phenomena occurring during drying remained similar for both laboratory and industrial scale [25].

**Figure 4:**
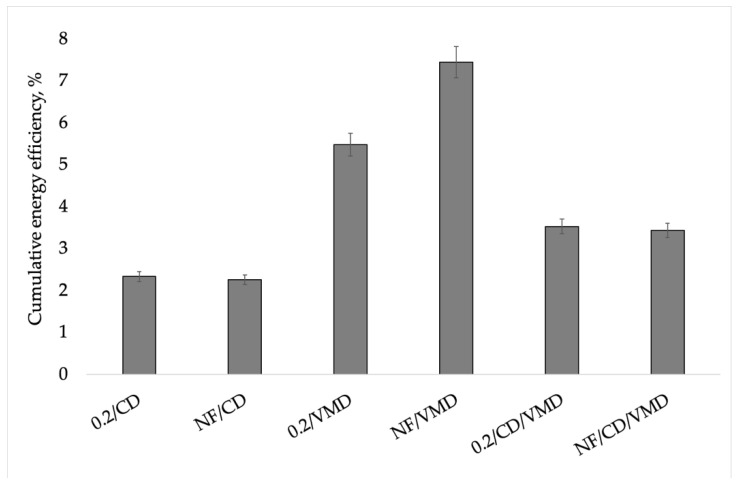
Cumulative energy efficiency during drying using different methods.

The cumulative energy efficiency during drying using different methods is shown in Figure 4. The highest values were obtained for the 0.2/VMD and NF/VMD variants (5.48 and 7.45%, respectively), which further support the high usefulness of the VMD method in the drying of apples dehydrated in chokeberry juice with the addition of mint extract. The lowest values were obtained for the samples dried by the CD method (2.34 and 2.26 for 0.2/CD and NF/CD, respectively), whereas combined drying proved to be slightly more effective (3.53 and 3.44 for 0.2/CD/VMD and NF/CD/VMD, respectively) in water evaporation compared to CD, which can be associated with the use of VMD as the finishing drying method. Considering previous studies [26], it can be assumed that the application of VMD at an earlier stage of drying could result in even higher energy efficiency, as VMD is more effective at water evaporation compared to CD.

Energy savings when CD/VMD or VMD were applied ranged from 53.2% to 80.4% compared to CD (Table 2). The use of VMD, either as a sole drying method or when combined with convective pre-drying, led to significant energy savings due to the higher drying constant *k* and shorter time (Table 2). However, the use of VMD comes with high investment costs that need to be considered when choosing the optimal drying method [29].

## 3. Materials and Methods

### 3.1. Materials

Commercial apples *cv. Champion* as well as mint plants were purchased on the local market (Wroclaw, Poland). Concentrated chokeberry juice (Rauch Polska, Płońsk, Poland) was filtrated prior to the experiment according to the method described by Masztalerz et al. [15] using a filter with 0.2 µm pore size. Apples of initial moisture content *M_C_* = 87.20% were cut into cylinders (18 ± 0.1 mm × 3.35 ± 0.15 mm).

### 3.2. Osmotic Dehydration

Osmotic dehydration was carried out using osmotic solutions (OS) prepared from filtrated and non-filtrated chokeberry juice with the addition of mint extract. Mint infusion was prepared by cutting the mint plants into small pieces and then putting them into the beakers with water (35 °C) for 1 h. The process was intensified by the use of ultrasounds during the first 5 min, which is supported by the previous studies by Masztalerz et al., who showed that the ultrasounds at the beginning of osmotic dehydration contribute to higher mass transfer [48]. Obtained infusion (30 mL) was then added to the concentrated chokeberry juice (120 mL). Osmotic dehydration was performed in water baths for 30 min using 30 g of apples and 150 mL of OS with the ultrasounds on during the first 5 min of the process. Osmotic dehydration was carried out in two technological repetitions.

### 3.3. Drying Methods

After osmotic dehydration, the samples were subjected to three drying methods, namely convective drying (CD), vacuum-microwave drying (VMD) and combined convective pre-drying followed by vacuum-microwave finishing drying (CD/VMD).

Convective drying was performed at 60 °C using a dryer constructed at the Institute of Agricultural Engineering (Wroclaw, Poland). VMD was carried out using an SM200 dryer at 480 W with a power reduction to 120 W when the temperature reached 80 °C in order to minimize the thermal damage to the sample due to overheating. The temperature was monitored using infrared camera i50 (Flir Systems AB, Stockholm, Sweden). Combined CD/VMD drying consisted of 60 min of CD at 60 °C and VMD at 480 W/120 W.

**Figure 5:**
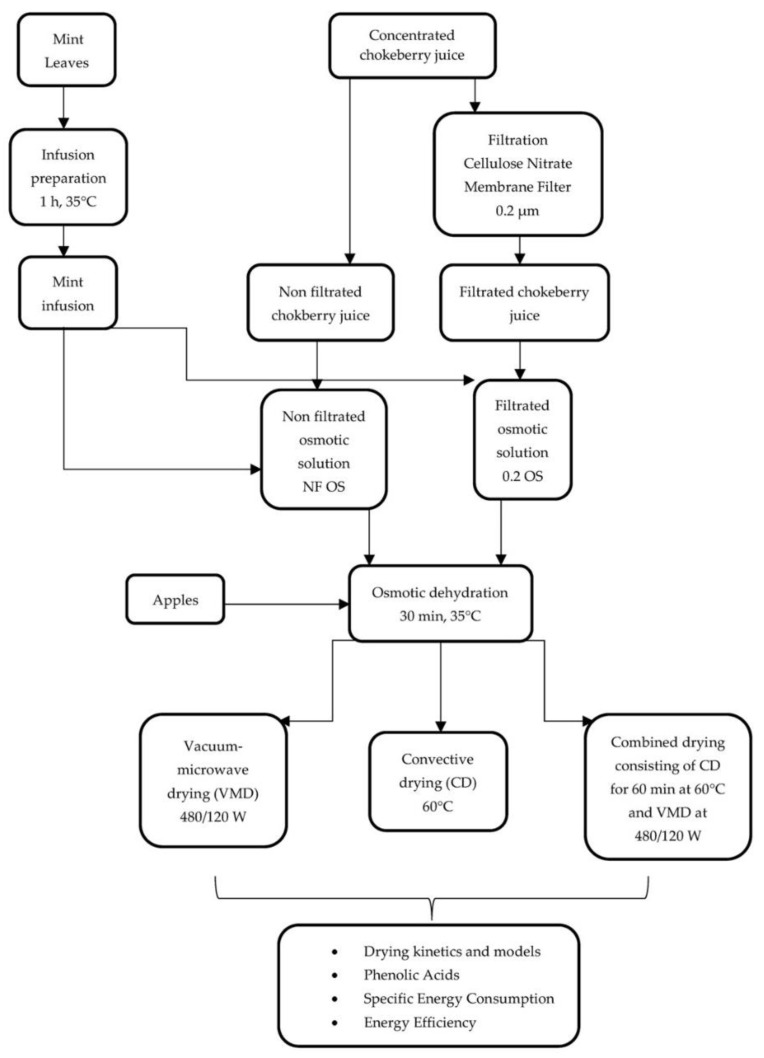
Process flow chart.

All drying methods were performed in two technological repetitions and the process was stopped when the moisture content of the samples was below 5%. Figure 5 shows the process flow chart.

Several models were used to fit the experimental data, namely, the Modified Page, Logarithmic and Henderson–Pabis models (Table 5).

### 3.4. Concentration of Osmotic Solutions

Concentration of the osmotic solutions (filtrated and non-filtrated solutions) was measured in three repetitions using Atago Digital Brix Refractometer PAL-3 (Atago Co., Ltd., Tokyo, Japan) and the results were expressed as °Brix.

### 3.5. Moisture Content

Moisture content of fresh and dried apples was obtained by drying the samples for 24 h at 70 °C under 100 Pa. The measurement was performed in 3 repetitions.

### 3.6. Color

Surface color of the samples was measured in five repetitions in reference to CIE L*a*b* color space using Chroma Meter CR-400 (Minolta Co., Ltd., Osaka, Japan). The lightness of the sample was determined based on the L* coordinate, where the higher the value the lighter the hue. Redness of the sample was shown using the a* (red-green) coordinate, whereas yellowness was presented as the b* (yellow-blue) coordinate. Total change in color in reference to the fresh material was determined as ∆E* according to Equation (1) provided by Cano-Lamadrid et al. [17]:

(1) $$∆E={\left[{\left(L-L^{*}\right)}^{2}+{\left(a-a^{*}\right)}^{2}+{\left(b-b^{*}\right)}^{2}\right]}^{0.5}$$

### 3.7. Energy Consumption

Energy consumption during osmotic dehydration and drying of the samples was calculated according to the Equations (1)–(5). The energy required for CD, VMD and CD/VMD was determined using the methodology described by Calín-Sanchez et al. [25]. Energy consumption during osmotic dehydration was estimated using Equation (2):

(2) $$E_{OD}=\left(N_{h}\cdot 0.2\cdot t_{OD}\right)+\left(N_{U}\cdot t_{u}\right)$$

where *N_h_* is the power of the heater installed in water baths, *t_OD_* is the time of osmotic dehydration, *N_U_* is the power of ultrasounds and *t_U_* is the time when the ultrasounds are switched on. The energy required for heating of the water inside the water batch was reduced to 20%, as after initial heating of the water, only a fraction of the power is needed to maintain the temperature.

Energy consumption during CD (*E_CD_*) was calculated using Equation (3):

(3) $$E_{CD}=\left(\frac{N_{f}}{6}+N_{h}\right)\cdot t$$

where *N_f_* is the power of the fan that is divided by six, which stands for the six drying columns that are fed by this fan, *N_h_* stands for the power of the heater and *t* is the time of drying.

Energy consumption during VMD (*E_VMD_*) was determined considering the output power of magnetrons (*N_M_*), working efficiency of magnetrons (*η_M_*), power of vacuum-pump (*N_V_*) and power of the electric engine used to rotate the sample (*N_e_*) (Equation (4)):

(4) $$E_{VMD}=\left(\frac{N_{M}}{\eta_{M}}+N_{V}+N_{e}\right)\cdot t$$

### 3.8. Specific Energy Consumption

The specific energy consumption was determined on the basis of Equations (5) and (6). In the case of combined drying, the specific energy consumption was calculated according to the equation provided by Chua et al. [29]:

(5) $$E_{CD-VMD}=\frac{E_{CD}+E_{VMD}}{m}$$

(6) $$E_{CD-VMD}=\frac{E_{CD}+E_{VMD}}{W}$$

where *m* means the initial mass (g) of the sample and *W* means the mass (g) of the water removed from the sample during drying.

### 3.9. Energy Efficiency

The energy efficiency (*η_d_*) was calculated using the ratio of the theoretical value of the energy (*E_t_*) required to evaporate free water from the material (Equation (7)) to the energy consumption needed in each process: *E_OD_* for osmotic dehydration, *E_CD_* for convective drying and *E_VMD_* in the case of vacuum-microwave drying:

(7) $$E_{t}=W\cdot \lambda_{w}$$

The latent heat of evaporation *λ_w_* was equal to 2.36 ± 0.02 kJ·g^−1^; the value was determined on the basis of the temperatures at the final stage of drying [25].

### 3.10. Energy Savings

Energy savings arising due to the application of VMD, both sole use or coupled use of VMD after convective pre-drying in the combined method (CD/VMD), were calculated with respect to CD using Equation (8) [26]:

(8) $$E_{s}=\frac{E_{CD}-E_{CD/VMD}}{E_{CD}}\;\cdot \;100\%$$

### 3.11. Chemical Analysis

#### 3.11.1. Sample Preparation

For solid sample preparation, samples (dehydrated apples) were extracted according to the method by Michalska et al. [51]. Briefly, 0.5 ± 0.1 g of sample was placed in a 20 mL vial with 10 mL of extraction mixture (methanol/water 80:20 [*v*/*v*]) and extracted by shaking (200 rpm) for 24 h in room temperature. Furthermore, 2 mL of extract was collected and centrifuged (13,000 rpm). The prepared samples were diluted 10 times (for caffeic acid and rosmarinic acid) or 100 times (for chlorogenic acid) before analysis.

For liquid samples (osmotic solutions), analysis was performed according to Wojdyło et al. [52]. Briefly, for the samples (chokeberry juice with mint infusion), 2 mL of liquid was collected and centrifuged (13,000 rpm). Thereafter, samples were diluted 10 times (for caffeic acid and rosmarinic acid) or 100 times (for chlorogenic acid) before analysis.

#### 3.11.2. LC-MS/MS Analysis

The separation and analysis were performed on LCMS-8045 apparatus (Shimadzu, Kyoto, Japan) equipped with a Kinetex 2.6u C18 100A 100 × 3.0 mm column with a SecurityGuard ULTRA 3 mm (Phenomenex, Torrance, CA, USA).

The mobile phase used for chromatographical separation consisted of 0.1% aqueous formic acid (A) and acetonitrile (B) (Sigma-Aldrich, St. Louis, MO, USA). The solvent gradient conditions were applied as follows: 2% to 20% B during 10 min; 20% to 60% B from 10 min to 15 min; and 60% to 2% B from 15 min to 20 min. The flow rate and injection volume were 0.3 mL/min and 10 μL, respectively. The column temperature was 40 °C. The operational conditions were obtained experimentally.

The mass spectrometry was performed using electrospray ionization (ESI) and multiple reaction monitoring (MRM) mode (detailed transitions data are given in Table 6) with negative ionization; nitrogen for nebulizing (flow 3 L/min) and desolvation (temperature 526 °C); both heating and drying gas flow at 10 L/min; interface, DL and heat block temperatures of 300, 250 and 400 °C, respectively. The optimal MS conditions were established via the “method optimalization” function.

The quantification of phenolic acids was done by comparison with the pure standards’ calibration curves. Pure standards of chlorogenic acid, caffeic acid and rosmarinic acid were obtained from Signa-Aldrich (Saint Louis, MO, USA). The standards’ solutions (in methanol) were prepared in concentrations of 0.00625 µg/mL, 0.0125 µg/mL, 0.025 µg/mL, 0.05 µg/mL, 0.5 µg/mL and 2.5 µg/mL.

### 3.12. Statistical Analysis

Mathematical models were fitted using Table Curve 2D v. 5.0 (Systat Software, Inc., San Jose, CA, USA) on the basis of the highest values of the coefficient of determination *R*^2^ and the lowest values of the root mean square error (*RMSE*). A one-way analysis of variance was applied to analyze the data using STATISTICA v. 13.0 (StatSoft, Inc., Tulsa, OK, USA).

The particle modeling and size approximation was obtained using HyperChem 8.07 (Hypercube Inc., Gainesville, FL, USA) according to the semi-empirical PM3 method (with set-up convergence limit 0.01, 50 iterations and a solvent box simulation with 220 water molecules and minimum distance 0.23 nm between the solvent particles and the soluble atom).

## 4. Conclusions

Filtration of osmotic solution made from concentrated chokeberry juice with the addition of mint infusion did not affect drying kinetics; however, it affected the selective penetration during osmotic dehydration. As a result, a higher number of phenolic acids after drying was found in the samples dehydrated in filtrated osmotic solution regardless of the applied drying method. Variants dried by convective drying (CD) had a higher content of caffeic acid (CA) and chlorogenic acid (ChA); however, they had a significantly lower content of rosmarinic acid (RA), whereas samples dried by vacuum-microwave drying (VMD) and combined methods (CD/VMD) had a significantly higher content of RA.

The color of the material after osmotic dehydration and drying was found to be changed to the color of chokeberry juice used during osmotic dehydration (OD), which shows that OD in concentrated juices can be used to naturally conceal discolorations occurring during drying. Drying kinetics of each drying method showed that the use of VMD significantly reduced the required time of drying. Page’s model was found to be the most suitable to fit the experimental data. The analysis of the specific energy consumption during drying showed that the time of the process had the highest influence on energy consumption; therefore, the best results were obtained for vacuum-microwave drying (VMD). However, the application of combined drying resulted in a significant reduction of the energy consumption compared to CD. Yet, the highest efficiency of water evaporation as well as the highest energy savings were reported for the VMD method. In conclusion, the best method considering energy consumption and phenolic acid content was VMD.

## Figures and Tables

**Table 1:** Solid gain (SG), water loss (WL), water loss to solid gain ratio (WL/SG) and specific energy consumption (*E_ODw_*) during osmotic dehydration in filtrated (0.2 OS) and non-filtrated (NF OS) osmotic solutions (OS).

	SG, g·g^−1^ fw	WL, g·g^−1^ fw	WL/SG	*E_ODw_*^,^ kJ·g^−1^ w	*E_ODm_,* kJ·g^−1^ fw
0.2 OS	0.050 ± 0.006 ^a,^^†^	0.218 ± 0.008 ^a^	4.228 ± 0.27 ^a^	578.67 ± 22.88 ^a^	3.54 ± 0.02 ^a^
NF OS	0.050 ± 0.002 ^a^	0.212 ± 0.021 ^a^	4.216 ± 0.34 ^a^	597.86 ± 60.54 ^a^	3.57 ± 0.02 ^a^

^†^ Values followed by the same letter, within the same column, were not significantly different (*p* < 0.05) according to Tukey’s HSD test.

**Table 2:** Mathematical models used to fit experimental data obtained during drying using different methods.

Model	Drying Conditions	Constants			Statistics	
		*A*	*k*	*B*	*RMSE*	*R* ^2^
Modified Page	0.2/CD	0.525	0.0157	1.12	0.0091	0.9972
	NF/CD	0.513	0.0118	1.19	0.0080	0.9978
	0.2/CD/VMD	0.122	0.5620	0.91	0.0051	0.9806
	NF/CD/VMD	0.141	0.4280	1.22	0.0054	0.9844
	0.2/VMD	0.527	0.1220	1.37	0.0094	0.9969
	NF/VMD	0.540	0.1070	1.43	0.0084	0.9972
		***A***	***k***	***C***	***RMSE***	***R*** **^2^**
Logarithmic	0.2/CD	0.538	0.0263	0.0047	0.0107	0.9962
	NF/CD	0.536	0.0261	0.0023	0.0126	0.9946
	0.2/CD/VMD	0.117	0.5790	0.0052	0.0013	0.9987
	NF/CD/VMD	0.137	0.5720	0.0041	0.0042	0.9906
	0.2/VMD	0.556	0.2280	−0.0064	0.0231	0.9812
	NF/VMD	0.576	0.2340	−0.0083	0.0262	0.9777
		***A***	***k***		***RMSE***	***R*^2^**
Henderson–Pabis	0.2/CD	0.541	0.0257		0.0109	0.9960
	NF/CD	0.538	0.0258		0.0124	0.9949
	0.2/CD/VMD	0.121	0.5120		0.0047	0.984
	NF/CD/VMD	0.141	0.5280		0.0052	0.9857
	0.2/VMD	0.551	0.2350		0.0223	0.9829
	NF/VMD	0.569	0.2320		0.0253	0.9794

NF OD—osmotic dehydration in non-filtrated osmotic solution; 0.2 OD—osmotic dehydration in filtrated (0.2 µm) osmotic solution; CD—convective drying; VMD—vacuum-microwave drying.

**Table 3:** The color parameters (L*, a*, b*, ∆E*) of the dried apples as well as the maximum temperature (*T_M_*), time of power reduction (*t_R_*), time of achieving 5% moisture content (*t_5%wb_*) and energy savings (*E_S_*) from the use of VMD for apple cylinders after drying using different methods.

Method	L*	a*	b*	∆E*	*T_M_*, °C	*t*, min		*t_R_*, min	*t_5%wb_*_,_ min		*E_S_,* %
						VMD	CD		CD	VMD	
Fresh	71.16 ± 3.21	−2.46 ± 0.45	10.62 ± 1.25	-	-	-	-	-	-	-	-
0.2/CD	25.49 ± 2.07 ^b,†^	14.59 ± 5.24 ^a,b^	1.51 ± 1.45 ^a^	49.6 ± 0.37 ^a^	60	360	-	-	285	-	-
NF/CD	24.1 ± 0.51 ^a,b^	8.58 ± 2.64 ^a^	0.22 ± 0.34 ^a^	49.45 ± 0.88 ^a^	60	360	-	-	288	-	-
0.2/CD/VMD	22.21 ± 0.25 ^a^	13.63 ± 2.76 ^a,b^	1.55 ± 0.83 ^a^	52.32 ± 0.85 ^b^	71	60	38	6	60	6 ± 1	53.2 ± 0.5 ^a^
NF/CD/VMD	23.39 ± 1.53 ^a,b^	16.86 ± 5.37 ^b^	2.12 ± 1.26 ^a^	52.22 ± 1.01 ^b^	70	60	38	6	60	6 ± 1	57 ± 0.4 ^b^
0.2/VMD	21.55 ± 1.13 ^a^	13.24 ± 2.53 ^a,b^	0.68 ± 0.64 ^a^	52.98 ± 0.77 ^b^	81	-	44	12	-	12 ± 1	80.4 ± 0.2 ^c^
NF/VMD	23.87 ± 1.52 ^a,b^	15.62 ± 3.08 ^a,b^	0.71 ± 0.91 ^a^	51.59 ± 0.65 ^b^	80	-	44	12	-	13 ± 1	80.2 ± 0.2 ^c^

^†^ Values followed by the same letter, within the same column, were not significantly different (*p* < 0.05) according to Tukey’s HSD test. NF OD—osmotic dehydration in non-filtrated osmotic solution; 0.2 OD—osmotic dehydration in filtrated (0.2 µm) osmotic solution; CD—convective drying; VMD—vacuum-microwave drying.

**Table 4:** Caffeic acid (CA), Rosmarinic acid (RA) and Chlorogenic acid (ChA) in the osmotic solutions before and after osmotic dehydration as well as in the material after drying using different methods.

**Apple**			
**Samples**	**CA** **mg per 100 g**	**RA** **mg per 100 g**	**ChA** **mg per 100 g**
CD NF	10.873 ± 0.970 ^b,c,^^†^	0.274 ± 0.065 ^a^	260.254 ± 9.391 ^a^
CD 0.2	11.205 ± 0.226 ^c^	0.193 ± 0.021 ^a^	236.512 ± 12.804 ^a,b^
CD/VMD NF	9.455 ± 0.138 ^a^	0.415 ± 0.034 ^b^	223.99 ± 7.238 ^b^
CD/VMD 0.2	9.592 ± 0.135 ^a^	0.438 ± 0.041 ^b^	222.477 ± 11.59 ^b^
VMD NF	9.864 ± 0.137 ^a,b^	0.422 ± 0.019 ^b^	219.658 ± 8.810 ^b^
VMD 0.2	10.505 ± 0.446 ^a,b,c^	0.432 ± 0.021 ^b^	225.255 ± 6.222 ^b^
**Osmotic Solution**			
**Samples**	**CA** **mg per 100 mL**	**RA** **mg per 100 mL**	**ChA** **mg per 100 mL**
NF + M 0’	4.382 ± 0.230 ^b^	9.847 ± 0.034 ^a^	179.076 ± 9.401 ^a^
NF + M 30’	2.509 ± 0.405 ^a^	7.333 ± 0.577 ^b^	131.140 ± 12.774 ^c^
0.2 + M 0’	4.713 ± 0.441 ^b^	4.434 ± 0.277 ^c^	162.352 ± 8.404 ^a,b^
0.2 + M 30’	3.998 ± 0.668 ^b^	3.331 ± 0.278 ^d^	148.615 ± 9.870 ^b,c^

^†^ Values followed by the same letter, within the same column, were not significantly different (*p* < 0.05) according to Tukey’s HSD test. NF OD—osmotic dehydration in non-filtrated osmotic solution; 0.2 OD—osmotic dehydration in filtrated (0.2 µm) osmotic solution; CD—convective drying; VMD—vacuum-microwave drying.

**Table 5:** Models used to describe drying kinetics.

Model	Model Equation	Reference
Modified Page	$MR=A\cdot e^{-k\cdot t^{B}}$	[26]
Logarithmic	$MR=A\cdot e^{-k\cdot t}+C$	[49]
Henderson–Pabis	$MR=A\cdot e^{-k\cdot t}$	[50]

**Table 6:** Parameters for marker compound quantification by MRM mode.

Compound	Precursor *m*/*z* [M − H]^−^	MRM Transitions *m*/*z* (Q_1_ → Q_3_)	Q1 Pre Bias(V)	Collision Energy	Q3 Pre Vias(V)
Caffeic acid	178.8000	178.8000→135.3000 178.8000→134.2500 178.8000→79.25000	20.0 20.0 20.0	18.0 26.0 24.0	26.0 25.0 14.0
Chlorogenic acid	353.0000	353.0000→191.3000 353.0000→85.2000 353.0000→93.2500	18.0 18.0 17.0	15.0 42.0 45.0	12.0 17.0 18.0
Rosmarinic acid	359.1000	359.1000→161.3000 359.1000→197.3000 359.1000→133.2500	18.0 18.0 18.0	16.0 17.0 40.0	20.0 12.0 26.0

## Data Availability

Data are available on request from the authors.

## Sample Availability

Samples of the compounds are not available from the authors.
